# RIBOSE PHOSPHATE ISOMERSASE 1 Influences Root Development by Acting on Cell Wall Biosynthesis, Actin Organization, and Auxin Transport in *Arabidopsis*

**DOI:** 10.3389/fpls.2019.01641

**Published:** 2020-01-08

**Authors:** Jia-Bao Huang, Yi Zou, Xiaojing Zhang, Mingyan Wang, Qingkun Dong, Li-Zhen Tao

**Affiliations:** ^1^ State Key Laboratory for Conservation and Utilization of Subtropical Agro-bioresources, South China Agricultural University, Guangzhou, China; ^2^ Guangdong Provincial Key Laboratory of Protein Function and Regulation in Agricultural Organisms, College of Life Sciences, South China Agricultural University, Guangzhou, China; ^3^ State Key Laboratory of Crop Biology, Shandong Agricultural University, Taian, China

**Keywords:** auxin, cell wall, embryo development, root development, RIBOSE PHOSPHATE ISOMERSASE 1 (RPI1)

## Abstract

Cell wall biosynthesis plays essential roles in cell division and expansion and thus is fundamental to plant growth and development. In this work, we show that an *Arabidopsis* mutant *dpr3*, isolated by a forward genetic screen, displays embryo defects and short, swelling primary root with the failure of maintenance of root apical meristem reminiscent to several cell wall–deficient mutants. Map-based cloning identified *dpr3* is a mutant allele of *RIBOSE PHOSPHATE ISOMERSASE 1* (*RPI1*), an enzyme involved in cellulose synthesis. Cellulose content in the mutant was dramatically decreased. Moreover, *dpr3* (*rpi1* from hereon) caused aberrant auxin distribution, as well as defective accumulation of root master regulators PLETHORA (PLT1 and PLT2) and misexpression of auxin response factor 5 (*MONOPTEROS, MP*). The abnormal auxin distribution is likely due to the reduced accumulation of auxin efflux transporters PIN-FORMED (PIN1 and PIN3). Surprisingly, we found that the orientation of actin microfilaments was severely altered in *rpi1* root cells, whereas the cortical microtubules stay normal. Our study provides evidence that the defects in cellulose synthesis *in rpi1* affect polar auxin transport possibly connected with altered F-actin organization, which is critically important for vesicle trafficking, thus exerting effects on auxin distribution, signaling, and auxin-mediated plant development.

## Introduction

Root growth depends on quiescent center (QC) and the surrounding stem cells which form the root stem cell niche (SCN) (Heidstra and Sabatini, 2014). All types of root cells originate from the SCN; thus, the maintenance of root SCN is critical for root growth (Scheres, 2007). There are two parallel pathways that had been identified for root SCN maintenance. The first one is involved in AP2 transcription factors PLETHORA (PLT), and the other one is associated with GRAS transcription factors SHORT-ROOT (SHR) and SCARECROW (SCR) (Nakajima et al., 2001; Sabatini et al., 2003; Aida et al., 2004; Galinha et al., 2007). The phytohormone auxin plays an important role in maintaining root SCN. Auxin response maximum in the distal stem cell region is required for QC function (Blilou et al., 2005). The transcription of *PLT* genes is dependent on auxin, and the auxin–PLT pathway acts as a core module in root stem cell maintenance and cell division for developing root (Galinha et al., 2007; Mahonen et al., 2014). *plt1plt2* double mutant showed strongly defective root SCN organization, giving rise to short root meristem phenotypes (Aida et al., 2004; Galinha et al., 2007).

Auxin distribution pattern acts as the developmental clue for growing plant, and the certain auxin pattern is mainly determined by polar auxin transport (PAT), which is mediated by polar located PIN-FORMED (PIN) efflux proteins on the plasma membrane (PM) to a great extent (Adamowski and Friml, 2015). Various regulators had been identified for the abundance and polarities maintenance of PINs, including the ARF-GTPase activator ARF-GEF (Kleine-Vehn et al., 2008a), AGCIII-type protein kinase PINOID (Friml et al., 2004), phosphatase 2A (Michniewicz et al., 2007), and D6 protein kinase and its family members (Zourelidou et al., 2014). Moreover, PINs undergo trafficking to the lytic vacuole for degradation, which is an important mechanism for maintaining the abundance of PIN proteins (Kleine-Vehn et al., 2008b; Nodzynski et al., 2013).

Other players are also involved in modulating PIN proteins. Firstly, studies have demonstrated the close correlation between cytoskeleton and PAT. Pharmacological investigations showed that treatments with microtubule (MT)-targeted drug oryzalin to depolymerize MTs reduced the basal distribution of PIN1 and PIN2 in root cells (Boutte et al., 2006; Kleine-Vehn et al., 2008b). The CLIP-ASSOCIATED PROTEIN (CLASP) mediates an association between PINs cycling and MTs by interacting with the retromer component sorting nexin 1. *clasp* mutants display a range of auxin-related phenotypes, including a reduction in root apical meristem size and increased lateral root abundance (Ambrose et al., 2007; Kirik et al., 2007; Ambrose et al., 2013). Several investigations confirmed actin cytoskeleton also links to the PAT. In *Arabidopsis*, actin-targeted drug latrunculin B inhibited intracellular PIN1 accumulation in brefeldin A (BFA) compartments and recycling of PIN1 to the PM after washout of BFA (Geldner et al., 2001). Latrunculin B treatment also caused PIN3 internalization in smaller compartments without any regular positioning in columella cells (Friml et al., 2002) and increased the [^3^H]indole-3-acetic acid (IAA) accumulation in *Fucus distichus* embryos (Sun et al., 2004). A study showed enhanced accumulation of the cortical fine actin of leaf epidermal cells inhibits clathrin-dependent PIN1 endocytosis, leading to enhanced PIN1 accumulation on the PM (Nagawa et al., 2012). In rice, defective F-actin arrays in *rmd (ROOT MORPHOLOGY DETERMINANT)* mutants disrupt expression of *OsPIN1b* and *OsPIN2*, auxin distribution, and auxin-mediated cell growth during root development (Li et al., 2014). Secondly, evidence was accumulating that the cell wall may function in the PAT. Treatment with cellulose synthesis inhibitor isoxaben led to hyperlocalization of PIN1 on the PM in shoot apical meristem (SAM) cells (Heisler et al., 2010). Genetic and pharmacological interference with cellulose synthesis led to enhanced lateral diffusion and reduced polarity of PIN2, indicating there is a connection between the cell wall and PIN polarity on the PM (Feraru et al., 2011). This connection was further confirmed by the finding that plant cell wall limits lateral diffusion of PM proteins (Martiniere et al., 2012). Hence, the tight link between the cell wall and PAT and, ultimately, plant SAM and root development has been proposed. Nevertheless, the molecular mechanism underlying, for now, remains largely elusive.

Ribose 5-phosphate isomerase (RPI) is a small group of enzymes which function in the oxidative pentose phosphate pathway (oxPPP), catalyzing the interconversion between ribose 5-phosphate (R5P) and ribulose 5-phosphate (Ru5P). There are four members in *RPI* gene family in *Arabidopsis thaliana*, RPI1–RPI4, with RPI1 and RPI2 showing cytosolic localization while the other two are located in plastids (Xiong et al., 2009). Genetic studies in *Arabidopsis* have revealed the role of *RPI1* and *RPI2* during plant development. *rsw10*, carrying a point mutation in *RPI1* gene, showed a lower level of cellulose and swelling root phenotypes under 31°C temperature condition (Howles et al., 2006). **RPI2* knockout plants appeared with defective chloroplast structure and reduced photosynthetic capacity.* When grown at a relative high temperature, the mutants presented premature cell death in the leaves (Xiong et al., 2009).

In this report, we isolated a *dpr3* mutant which is a novel allele of *RPI1* gene. *dpr3* displays short, swelling roots and abnormal cell divisions in the basal region of embryos in *Arabidopsis*, indicating *RPI1* is required for proper embryo and root development. Further studies revealed the mutation in *RPI1* gives rise to altered auxin distribution and defective auxin-dependent PLT1 and PLT2 accumulation as well as *MP* expression. By analyzing auxin transport markers, we found the aberrant auxin distribution in the mutant and the reduced abundance of auxin efflux proteins PIN1 and PIN3 on the PM. Moreover, the *rpi1* presented a more transverse F-actin array rather than longitudinal aligned in root cells. Our results suggest a role of *RPI1* for actin cytoskeleton may link cell wall and PAT in the coordination of auxin-dependent root cell growth and patterning.

## Methods

### Plant Materials and Growth Conditions

*A. thaliana* Columbia-0 (Col-0) accession was used in this research. The *DR5_rev_:GFP* and *PIN1_pro_:PIN1-GFP* (Benkova et al., 2003), *PIN2_pro_:PIN2-GFP* (Xu and Scheres, 2005), *PIN3_pro_:PIN3-GFP* (Dello Ioio et al., 2008), *PLT1_pro_:PLT1-YFP*, *PLT2_pro_:PLT2-YFP* (Grieneisen et al., 2007), *WOX5_pro_:GFP* (Sarkar et al., 2007), *MP_pro_:n3xGFP* (Rademacher et al., 2012), *SHR_pro_: SHR-GFP* (Nakajima et al., 2001), *SCR_pro_ : GFP* (Wysocka-Diller et al., 2000), *35S_pro_ : Actin-binding Domain 2* (*ABD2*)*–GFP* (Wang et al., 2008), and *35S_pro_ : GFP-tubulin* (Bannigan et al., 2006) marker lines have been described before. Surface-sterilized seeds were sowed on 1/2 Murashige and Skoog (MS) medium (1% sucrose, 0.8% agar) and then followed by cold-treated at 4°C for 3 days in darkness and transferring to a phytotron set (light:dark = 16:8 h, 70% humidity, 22 ℃) The marker lines were individually crossed into *rpi1* mutant. Homozygous hybrid lines were obtained in F_2_ populations and analyzed in F_3_ or F_4_ generations.

### Root Phenotypic Analysis

Seedlings were grown on 1/2 MS standard medium for 2–10 days. The primary root length and meristem cell length were measured using Image J software. The meristem length, which is determined by the number of cortical cells from the stem cell to the first elongated cell, was investigated after soaking in HCG solution (Dello Ioio et al., 2007). Data presented were means with SD of 30 to 40 seedlings. For genetic complementation tests, the primary root and meristem length were analyzed after 10 days. For chemical complementation, seedlings of Col-0 and *dpr3* were grown on 1/2 MS standard medium with or without 2.5 mM uridine supplemented for 9 days, and the primary root and meristem length were measured. For 2,6-dichlorobenzonitrile (DCB; Sigma-Aldrich) treatment, seedlings of wild type bearing *PIN1_pro_:PIN1-GFP*, *PIN3_pro_:PIN3-GFP*, and *35S_pro_ : ABD2-GFP* markers, respectively, were grown on 1/2 MS standard medium with or without 0.1 μM of DCB (20 mM DCB stock solution, dissolved in DMSO).

### Map-Based Cloning of *DPR3*

The EMS-mutagenized *Arabidopsis* plant *dpr3* (Col-0) was crossed with Landsberg *erecta* (L*er*) accession. And 40 *dpr3* × L*er* F_2_ plants of the *dpr3* (short root) phenotype were used for rough mapping. Positional mapping showed that the site of *dpr3* mutation was flanked by two simple sequence length polymorphism (SSLP) markers (within BACs F10D13 and F10A5 respectively) on the lower arm of chromosome 1. Additional 98 short root F_2_ plants were analyzed to be narrowed down to a 670 kbp region (26.60 to 27.36 Mbp). Candidate genes in this region were PCR-amplified and sequenced, and a C-to-T transition resulting in the predicted Ala113Val amino acid change in the locus At1g71100 (RPI1) was found to be the casual single nucleotide polymorphism (SNP).

### Vector Construction and Plant Transformation

To construct *rpi1/RPI1_pro_:RPI1-GFP*, the *RPI1* promoter and coding sequence (CDS) were amplified by PCR from Col-0 genomic DNA or cDNA templates respectively and then inserted into the binary vector pCAMBIA1300, together with in-frame fused GFP. *Agrobacterium tumefaciens* (C58) with the resulting plasmid was introduced into *rpi1* mutant background using the floral dipping method (Clough and Bent, 1998). The relevant primer sequences were listed in **Table S1**. PCR primers were designed with the Primer Premier 5.0 software.

### Expression Pattern and Subcellular Localization Analysis of RPI1

The embryos and 4-day-old seedlings of transgenic *rpi1/RPI1_pro_:RPI1-GFP* plants were used for gene expression pattern analysis and subcellular localization analysis by confocal imaging.

## qRT-PCR Analysis

Whole roots of 6-day-old seedlings were used for total RNA preparation by using the plant RNA extraction kit (Huayueyang, Beijing, China). cDNA was synthesized by PrimeScript reverse transcriptase (TakaRa, Kusatsu, Shiga, Japan) from 3 μg of total RNA. PCR reaction was carried out using Illumina Eco system (Illumina, San Diego, California, USA) with a SYBR green probe (Vazyme, Nanjing, China). Expression of *PIN1* and *PIN3* in *rpi1* and wild type plants was normalized to the internal control *ACTIN2*, respectively. Three biological replicates were performed, and data presented are means with SD. Related primers used are listed in **Table S1**.

### Cellulose Measurement

Cellulose content measurement was performed according the method described by Bailey (1958) with minor modification. Briefly, 200 mg of 8-day-old dried seedlings was boiled in acetic acid and nitric acid for 25 min. The sample was then filtered, and 5 ml of this filtrate was mixed with 95 ml ddH_2_O for 1:20 dilution. Two milliliters of this dilution was used to perform colorimetric determination by adding 0.5 ml anthrone solution (2 g of anthrone in 100 ml of ethyl acetate) and 5 ml sulfuric acid was added to mix well and sit at room temperature for 12 min. The mixture was then subjected to a spectrophotometer (MAPADA^®^ V1300, Shanghai, China) assay at the wavelength of 625 nm. The content of cellulose was calculated according to the glucose stand curve.

### Microscopy

Ovules were cleared in Hoyer’s solution and roots in HCG solution as previously described (Chen et al., 2011). Differential interference contrast (DIC) pictures were taken on a Olympus BX51 microscope (Tokyo, Japan) connected with a Qimaging Ritiga 2000R digital camera (Surrey, BC, Canada). FM4-64 (5 μM) or propidium iodide (100 μg/ml) was used to visualize cell contour. Samples were imaged under ZEISS confocal microscopy (LSM 780) with excitation/emission (Ex/Em) wavelengths: GFP (488/505~530 nm), YFP (514/530~560 nm), FM4-64 (543/600~ nm), propidium iodide (561/591~635 nm), and kFluor615 (560/645~ nm). Twenty to 30 samples were examined for each group, and 15 to 20 samples were used for confocal imaging afterward. Similar results were obtained in three independent experiments.

### Accession Numbers

ACT2 (At3g18780), PIN1 (At1g73590), PIN2 (At5g57090), PIN3 (At1g70940), PLT1 (At3g20840), PLT2 (At1g51190), RPI1 (AT1G71100), SCR (At3g54220), SHR (At4g37650), WOX5 (At3g11260).

## Results

### *DPR3* Is Required for Maintenance of Root Meristem Development

**Figure 1:**
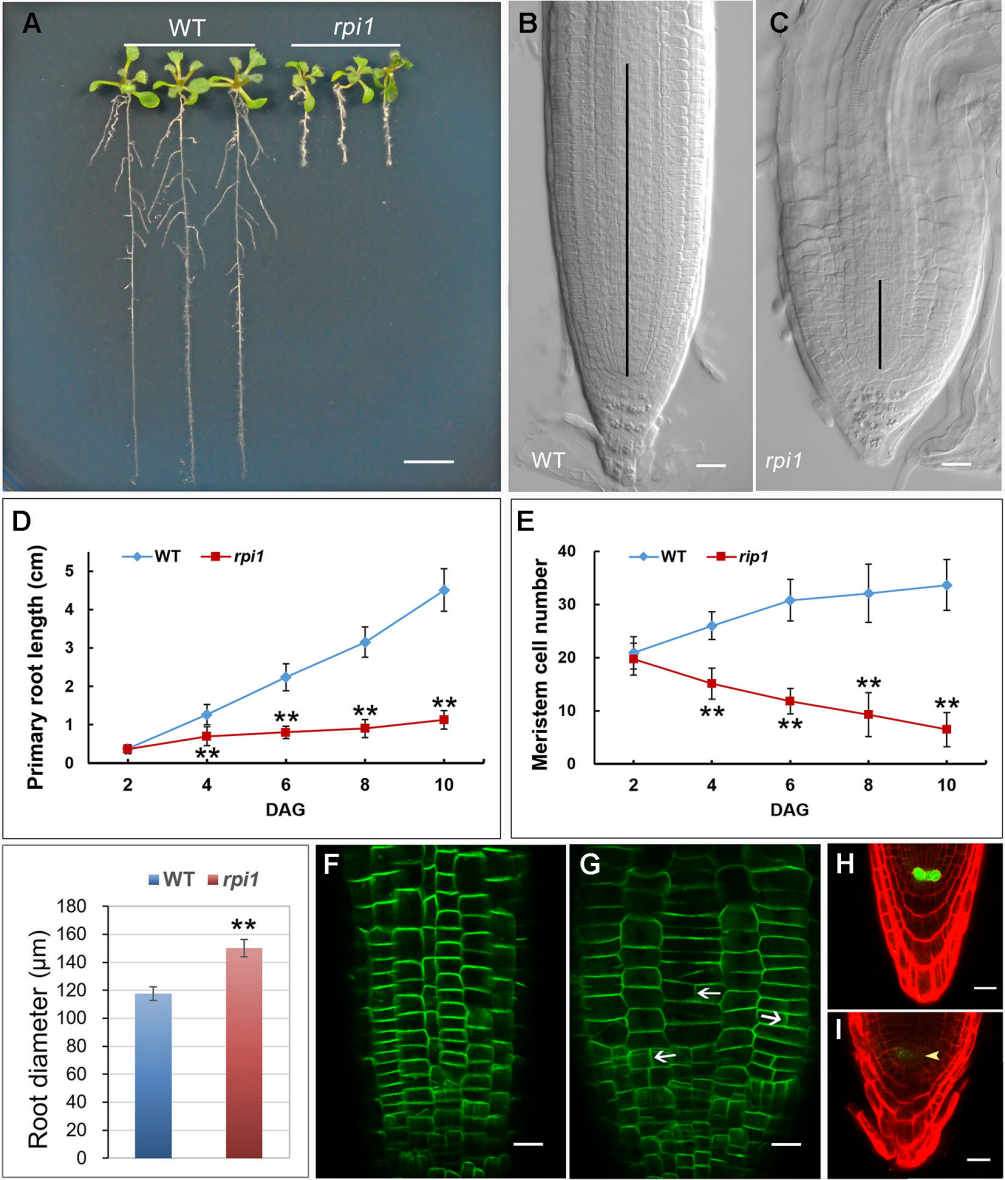
Phenotypes of dpr3 mutant roots. **(A)** Phenotype of the wild type (WT, left) and dpr3 (right) seedlings at 10 days after germination (DAG). **(B**, **C)** Root tips of the wild type (b) and dpr3 (c) at 10 DAG. The black vertical lines indicate the root meristem region. **(D)** Primary root length of wild type and dpr3 seedlings from 2 to 10 DAG. Data shown are average and SD (n = 30). Asterisks indicate Student’s t-test significant difference (**P < 0.01). **(E)** Root meristem cell number of the wild type and dpr3 on 2 to 10 DAG. The root meristem cell number is designated as the number of cortex cells in the cortex file extending from the quiescent center (QC) to the transition zone. Data shown are average and SD (n = 30). Asterisks indicate Student’s t-test significant difference (**P < 0.01). **(F)** Diameters of primary root meristem. The diameters were measured where the transition zone appeared. Data shown are average and SD (n = 30). Asterisks indicate Student’s t-test significant difference (**P < 0.01). **(G, H)** The expression of PIN2pro:PIN2-GFP in wild type (g) and dpr3 (h) root tips at 6 DAG. White arrowheads indicate the abnormal cell plates. **(I, J)** The expression of WOX5pro:GFP in roots of 4-day-old wild type **(I)** and dpr3 mutant seedlings **(J)**. Bars = 5 mm in **(A)**; 20 μm in **(B**,**C**,**G**–**J)**.

One mutant showing significantly short primary root was isolated from an ethyl methane sulfonate (EMS)–mutagenized population of *Arabidopsis*. The mutant, hereafter referred to as *defective primary root 3* (*dpr3*), showed stunted root growth (**Figure 1A**) and reduced meristem length (**Figures 1B**, **C**). Primary root growth was markedly reduced in *dpr3* from as early as 4 days after germination (DAG), compared with wild type (**Figure 1D**). And the number of meristematic cells significantly reduced over time in the root of *dpr3* (**Figure 1E**). Besides, *dpr3* mutant had a large diameter of roots (**Figure 1F**) and show swelling phenotype grown under normal condition (**Figure 1C**). Moreover, some meristematic epidermal cells showed vertical cell division plane (12%, n = 15) in *dpr3* roots, in comparison to the horizontal division plane in wild type (**Figures 1G**, **H**). These results indicate that DPR3 plays an important role in root meristem pattern formation in *Arabidopsis*.

*WUSCHEL-RELATED HOMEOBOX 5 (WOX5)* is mainly required for root columella stem cell activity (Sarkar et al., 2007). We investigated whether *WOX5* is affected in *dpr3* through examining the expression of the *WOX5_pro_:GFP* (Blilou et al., 2005; Sarkar et al., 2007). The intensity of *WOX5_pro_:GFP* was significantly reduced in the QC of *dpr3* roots, compared with wild type roots (**Figures 1I**, **J**). We also examined the *WOX5* expression in the embryos and similar results were observed (**Figure S1**).

### *DPR3* Shows Aberrant Cell Divisions in Embryo Development

**Figure 2:**
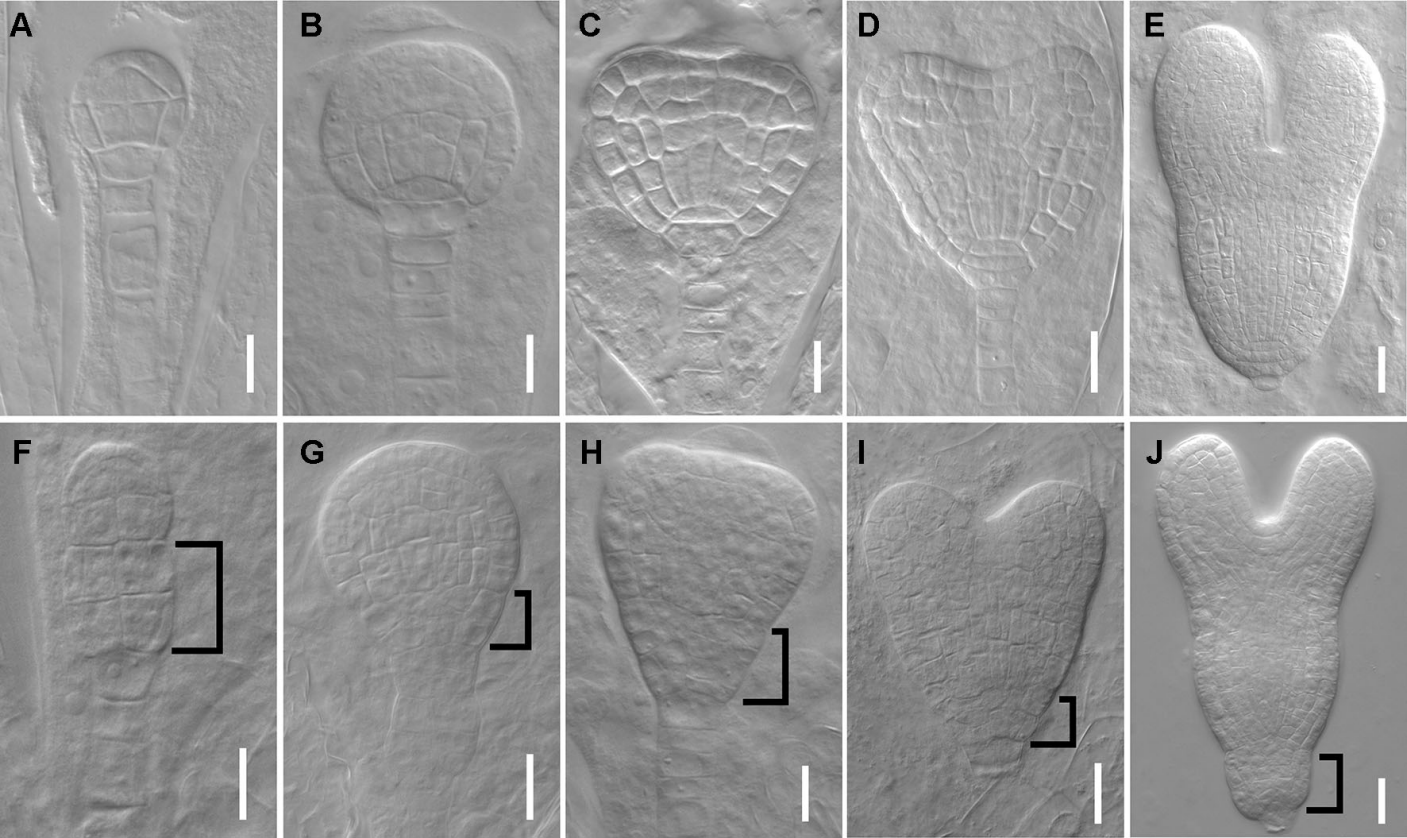
dpr3 mutants have defective cell divisions in the root pole of embryos. **(A**–**E)** Wild type embryos at 16-cell **(A)**, globular **(B)**, triangle **(C)**, heart **(D)**, and later heart **(E)** stages. **(F**–**J)** Embryos of dpr3 mutants at 16-cell **(F)**, globular **(G)**, triangle **(H)**, heart **(I)**, and later heart **(J)** stages. Bracketed area displays cell division defects in basal embryo region. Bars = 20 μm in **(D**, **E**, **I**, **J)** and 10 μm for the rest of the images.

We next tested whether the mutant has defects in embryos since the root SCN is established at embryo stage. At 16-cell stage, the hypophysis cell was specified, generating a normal basal pole in wild type embryo (**Figure 2A**), whereas the *dpr3* embryo displayed unclear boundary of the apical and basal pole (**Figure 2F**). From the globular stage onward, *dpr3* embryos showed frequent cell division defects in embryonic root pole (**Figures 2G–J** compared with **Figures 2B–E**; also see **Table S2**). Besides, excessively dividing suspensor cells were observed in some individuals (**Figure 2G**), in contrast to wild type (**Figure 2B**). Taken together, these data demonstrate that DPR3 is important for promoting the normal pattern formation during embryonic root development.

### Map-Based Cloning of *DPR3* Gene

Using positional cloning, we first mapped the *dpr3* locus on the short arm of chromosome 1, flanking by markers F10D13 and F10A5 in a physical region of ~3 Mb. Fine mapping was performed and localized the mutation in a region between 26.69 and 27.36 Mb. Sequencing analysis showed a point mutation occurring in a gene coding for *RIBOSE-5-PHOSPHATE ISOMERASE 1* (*RPI1*) in *Arabidopsis* (**Figure S2A**). This point mutation involves substitution of C to T at 338 bp, leading to amino acid transition of Ala113Val (**Figure S2B**), which is different from the previously reported *rsw10* (Glu115Lys) mutant of RPI1, which showed the temperature-dependent swelling root and a significant reduction in cellulose content (Howles et al., 2006).

To verify whether the *dpr3* phenotypes are caused by *RPI1* deficiency, a vector carrying *RPI1_pro_:RPI1-GFP* was introduced into *dpr3* mutant and the phenotypes of transgenic plants were analyzed. The recovered root and meristem length of these *dpr3*/*RPI1_pro_:RPI1-GFP* plants indicate that introduction of *RPI1* into the mutant background can rescue the mutant phenotypes (**Figures S3A**, **D**–**G**, **L**, **M**), including their embryo defects (**Figure S4** and **Table S2**). RPI1 catalyzes ribose interconversion, which is required for the synthesis of ribonucleotides. Furthermore, the mutant phenotype was complemented by exogenous application of uridine, which was one component of cellulose biosynthesis substrate, uridine 5’-diphosphate–glucose (UDP–glucose), as suggested in the study of *rsw10* (Howles et al., 2006; McFarlane et al., 2014). The *dpr3* mutant showed significantly shorter root and meristem lengths, compared with wild type (**Figures S3B**, **H**, **I**, **N**, **O**), and these defects were rescued while growing on the medium supplemented with 2.5 mM uridine (**Figures S3C**, **J**, **K**, **N**, **O**). Together, these data show that the developmental aberrations of *dpr3* can be well restored *via* genetic and chemical complementation, revealing the role of *RPI1* in maintaining normal root development.

To further understand the *RPI1* function, we investigated its expression pattern and subcellular localization using *rpi1/RPI1_pro_:RPI1-GFP* transgenic plants. We found that RPI1 was expressed in embryos and multiple organs of seedling, root, and also other organs like hypocotyl and leaf (**Figure S5**). The cytosolic subcellular localization of RPI1 was clearly observed by getting closer look to the GFP signal in root meristem cells (**Figure S5I**).

### Mutation in RPI1 Affects the Expression of Auxin Reporter Gene

**Figure 3:**
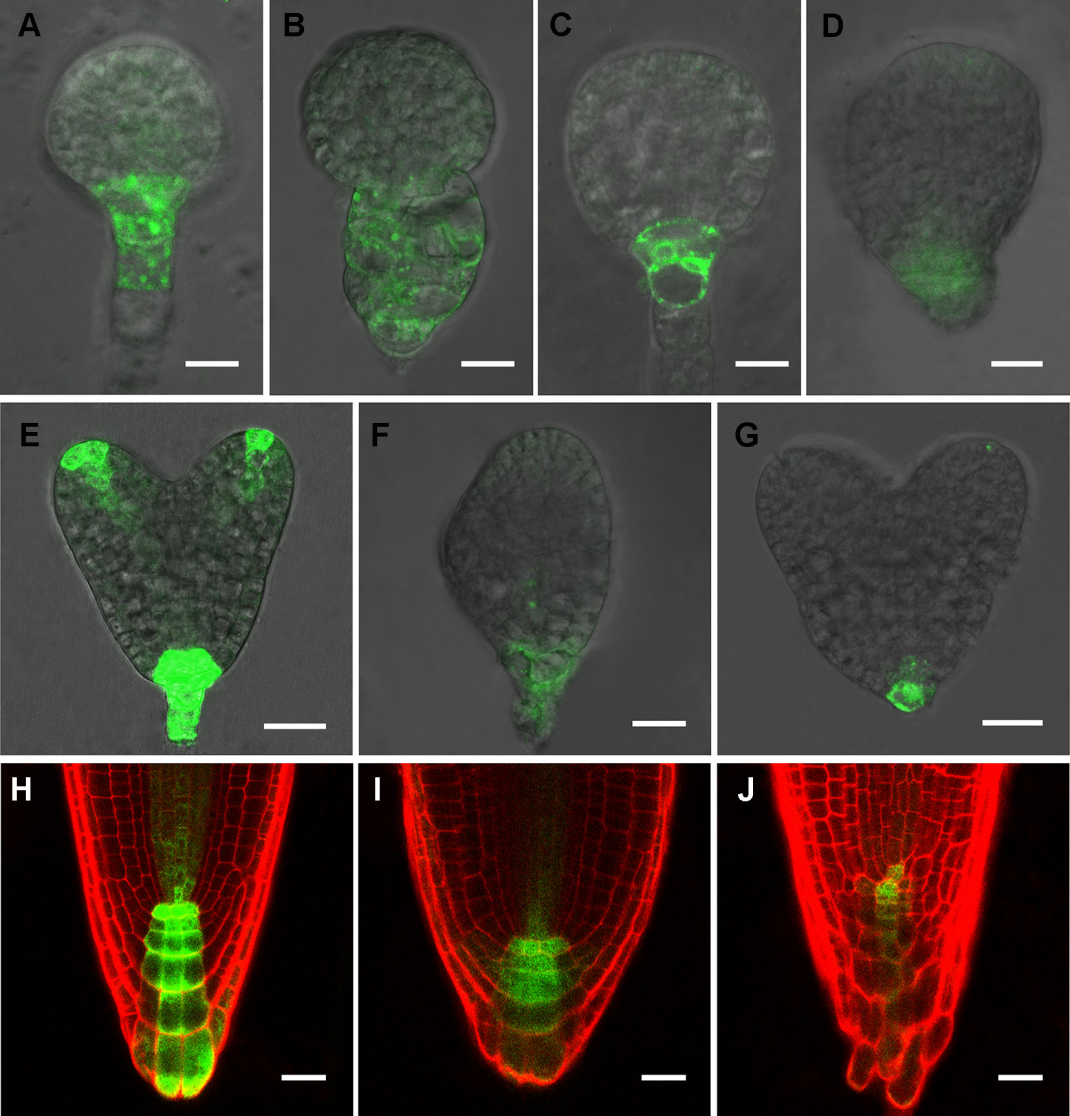
RIBOSE PHOSPHATE ISOMERSASE 1 (RPI1) is required for auxin maximum maintenance. **(A**–**G)** DR5_rev_:GFP expression in embryos at early globular, later globular, and heart stages of the wild type **(A, C, E)** and rpi1 mutant **(B, D, F, G).** **(H**–**J)** DR5rev:GFP expression in roots of 4-day-old wild type **(H)** and rpi1 **(I, J)** seedlings. Bars = 10 μm in **(A**–**D)** and 20 μm for the rest of the images.

Phenotypic analyses suggest that auxin-regulated processes could be affected in *rpi1* mutants (**Figures 1** and **2**). As indicated by the auxin reporter *DR5_rev_:GFP*, an auxin maximum was observed at the embryonic root pole of wild type (100%, n = 30; **Figures 3A**, **C**, **E**). In contrast, altered auxin maximum was detected in a majority of (77%, n = 31) *rpi1* embryos (**Figures 3B**, **D**, **F**, **G**). We then checked DR5 activity in the roots of *rpi1* seedlings. Comparing with the wild type (**Figure 3H**), the expression of *DR5_rev_:GFP*, auxin reporter was suppressed in *rpi1* mutant (**Figures 3I**, **J**). These data suggested that auxin maximum was perturbed in *rpi1* embryos and roots, which correlated with the observed abnormal cell divisions in the embryonic root pole and retarded root meristem development.

### *rpi1* Exhibited Altered Expression Pattern of PLT1/PLT2 and *MP*

**Figure 4:**
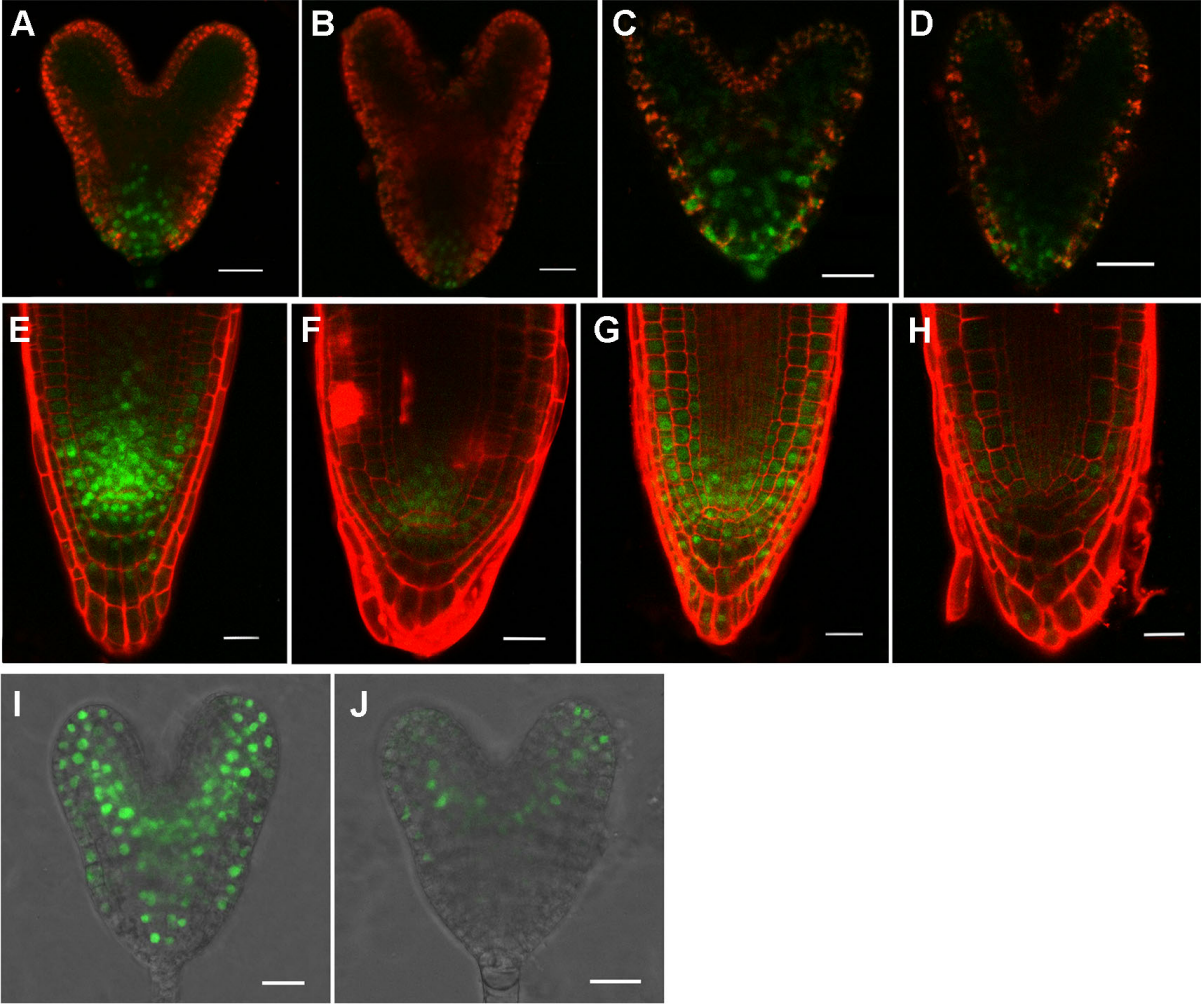
rpI1 mutation affects the expression of PLETHORA (PLT1/PLT2) and MONOPTEROS (MP) in embryos and roots. **(A**, **B)** The expression of PLT1pro:PLT1-YFP in embryos of the wild type **(A)** and rpi1 mutant **(B)**. **(C, D)** The expression of PLT2pro:PLT2-YFP in embryos of the wild type (c) and rpi1 mutant **(D)**. **(E**, **F)** The expression of PLT1pro:PLT1-YFP in 4-day-old roots of the wild type **(E)** and rpi1 mutant **(F)**. **(G**, **H)** The expression of PLT2pro:PLT2-YFP in 4-day-old roots of the wild type **(G)** and rpi1 mutant **(H)**. **(I, J)** MPpro:3XGFP is expressed in the embryos of wild type **(I)** and rpi1 **(J)** at heart stages. Bars = 20 μm.

The above data suggested that auxin was involved in causing the defects in *rpi1*. We then asked whether the expression of auxin-induced regulators PLT1/PLT2, which were well known for controlling cell fate specification, were affected in *rpi1*. We found both PLT1 and PLT2 were down-regulated in *rpi1* embryos and roots (**Figures 4B**, **D**, **F**, **H** compared with **Figures 4A**, **C**, **E**, **G**). *MONOPTEROS* (*MP*/*ARF5*) is a key transcription factor in auxin signaling in the embryo (Weijers et al., 2006). We next aimed to ascertain whether *MP* expression was affected. Strong *MP_pro_:n3XGFP* signal was detected in the pro-vascular tissue of heart stage embryos (**Figure 4I**), the expression of *MP* was largely altered in the *rpi1* mutant embryos (**Figure 4J**). The SHR-SCR pathway acts parallel with PLTs for the specification and maintenance of the root SCN and root growth (Helariutta et al., 2000; Sabatini et al., 2003; Aida et al., 2004). In contrast to PLT1/PLT2, the localization patterns of SHR and SCR were not altered in *rpi1* mutants (**Figure S6**). These results suggest that the defects observed in *rpi1* embryos and roots might result from aberrant expression pattern of PLTs and *MP*.

### Mutation of RPI1 Affects the Accumulation of PIN1 and PIN3

**Figure 5:**
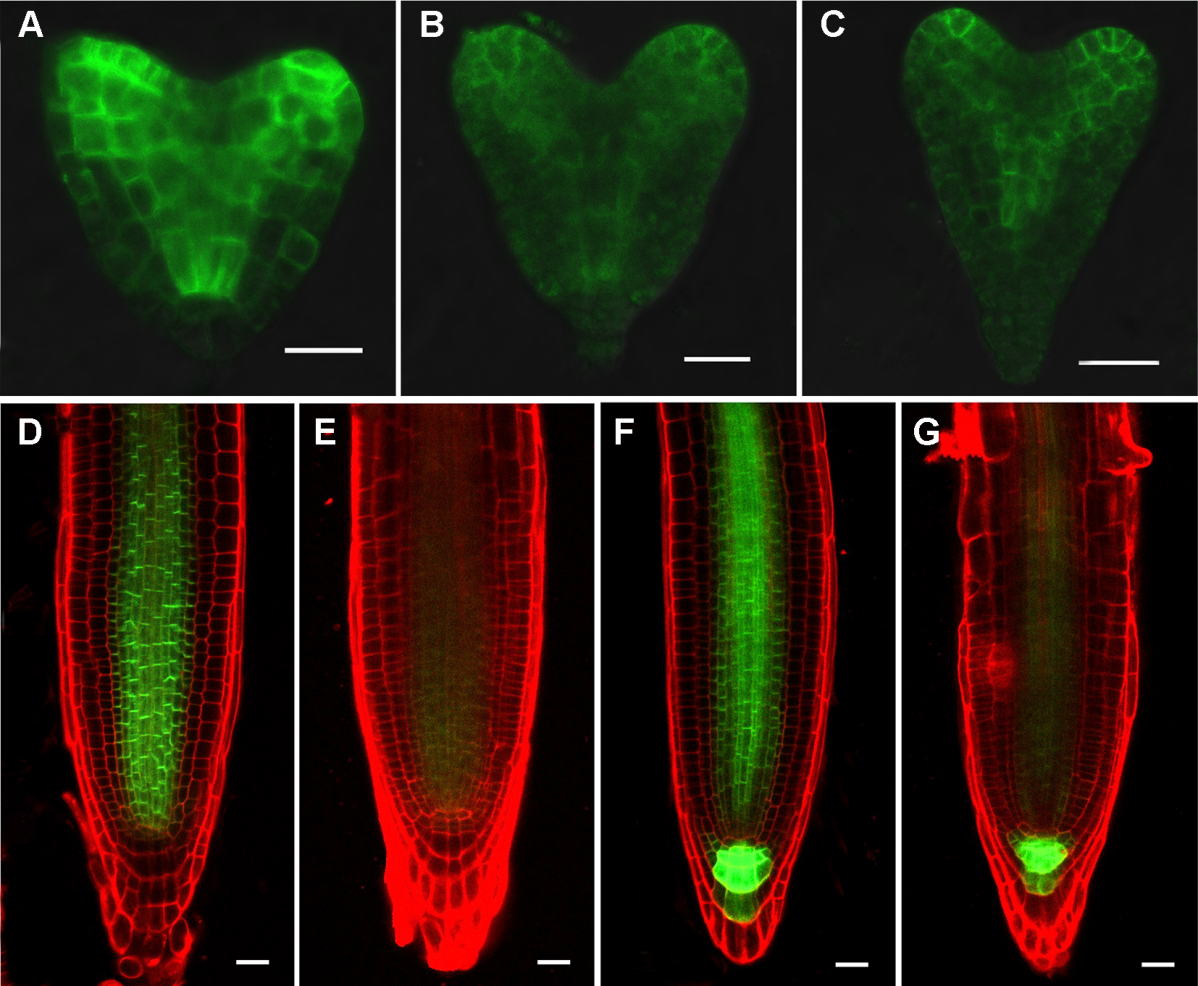
*RPI1* mutation reduces the expression of PIN1 and PIN3. **(A**–**C)** *PIN1pro:PIN1-GFP* is expressed in embryos of the wild type **(B)** and rpi1 mutant **(B, C)** at heart stages. **(D, E)** *PIN1pro:PIN1-GFP* expression pattern in 4-day-old wild type **(D)** and rpi1 **(E)** root tips. (F, G) *PIN3pro:PIN3-GFP* expression pattern in 4-day-old wild type **(F)** and rpi1 **(G)** root tips. Bars = 20 μm.

We next set out to determine whether the reduced DR5 activity in embryos and root tips in *rpi1* mutants is associated with an alteration in auxin transport.** We first examined the level of PIN1 in the mutant. At heart stage of embryo development, strong and basal localization of PIN1-GFP was observed in wild type (**Figure 5A**), whereas there were 80% (n = 30) of *rpi1* embryos, which displayed reduced PIN1-GFP accumulation in the corresponding regions (**Figures 5B**,** C**). In wild type seedling root tips, PIN1-GFP was expressed in the stele and endodermis (**Figure 5D**). In contrast, PIN1-GFP was strongly suppressed in the *rpi1* roots (**Figure 5E**). Then we analyzed the expression of other auxin transporters and found that PIN3-GFP signal was faint in *rpi1* root stele cells (**Figure 5G**) compared to its amount in wild type roots (**Figure 5F**). These observations suggest that perturbed auxin distribution in *rpi1* embryos (**Figures 3B**,** D**,** F**,** G**) and roots (**Figures 3I**, **J**) could result from the altered accumulation of PIN1 and PIN3**. However, we confirmed that mutation in *RPI1* did not result in changes in *PIN1* and *PIN3* at the transcriptional level in roots (**Figure S9**).

### *rpi1* Showed Abnormal Cellulose Synthesis and Altered Cortical Actin Filament Orientation

**Figure 6:**
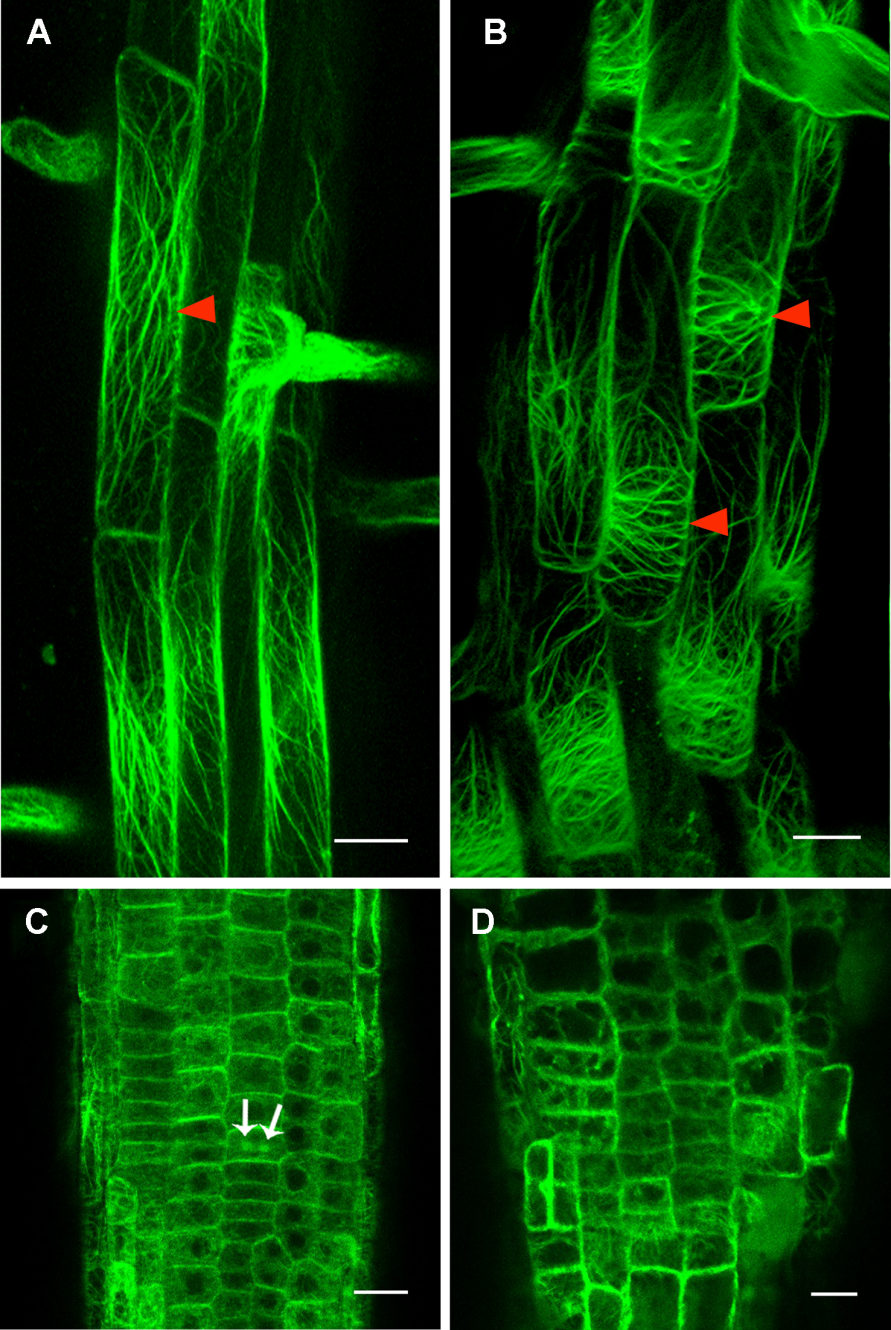
Cortical actin filament orientation is affected in rpi1 mutant. **(A)** Cortical actin filaments are mainly longitudinal along the direction of elongation in 4-day-old control seedling root cells (arrowheads). Each figure is a maximum projection of five slices z-stack. **(B)** Cortical actin filaments are transversely oriented in root cells of 4-day-old rpi1 seedling (arrowheads). Each figure is a maximum projection of five slices z-stack. **(C, D)** Cortical actin filaments are randomly oriented in the meristem zone in **(C)** wild type and **(D)** rpi1. Arrows indicate phragmoplast-enriched actin filaments. Bars = 50 μm in **(A, B)** and 20 μm in **(C, D**).

Our investigation demonstrated a severely decreased cellulose content in *rpi1* mutant (**Figure S7**), implying the cell wall architecture was largely affected. Previous studies have shown the cellulose deficiency could lead to the altered polarity of PIN proteins in root cells (Feraru et al., 2011) and disturbed cytoskeleton organization (Panteris et al., 2013; Paredez et al., 2008; Peng et al., 2013). To investigate the role of the cytoskeleton, we first explored whether the mutation in *RPI1* had an effect on the MT cytoskeleton using a maker line expressing *35S_pro_ : GFP-tubulin*, and we found that the MT arrays were not affected in root mature zone and meristem region of the mutant (**Figure S8**). Then we tested the actin cytoskeleton. As revealed by an actin marker, *35S_pro_ : ABD2-GFP*, actin filaments were mainly longitudinal oriented in wild type root cells in the elongation and differentiation zone (**Figure 6A**). By contrast, they showed transversely oriented pattern in the cells of the same zone in *rpi1* root (**Figure 6B**). In the meristematic region of the root, actin filaments orient randomly both in wild type and *rpi1* (**Figures 6C**, ** D**), reflecting an isotropic manner of cell enlargement. And in wild type roots, we could observe ABD2-GFP–labeled phragmoplasts, indicating active cell division events (**Figure 6C**). These results demonstrate that RPI1 is required for the normal F-actin organization in root but not for the regulation of MTs.

**Figure 7:**
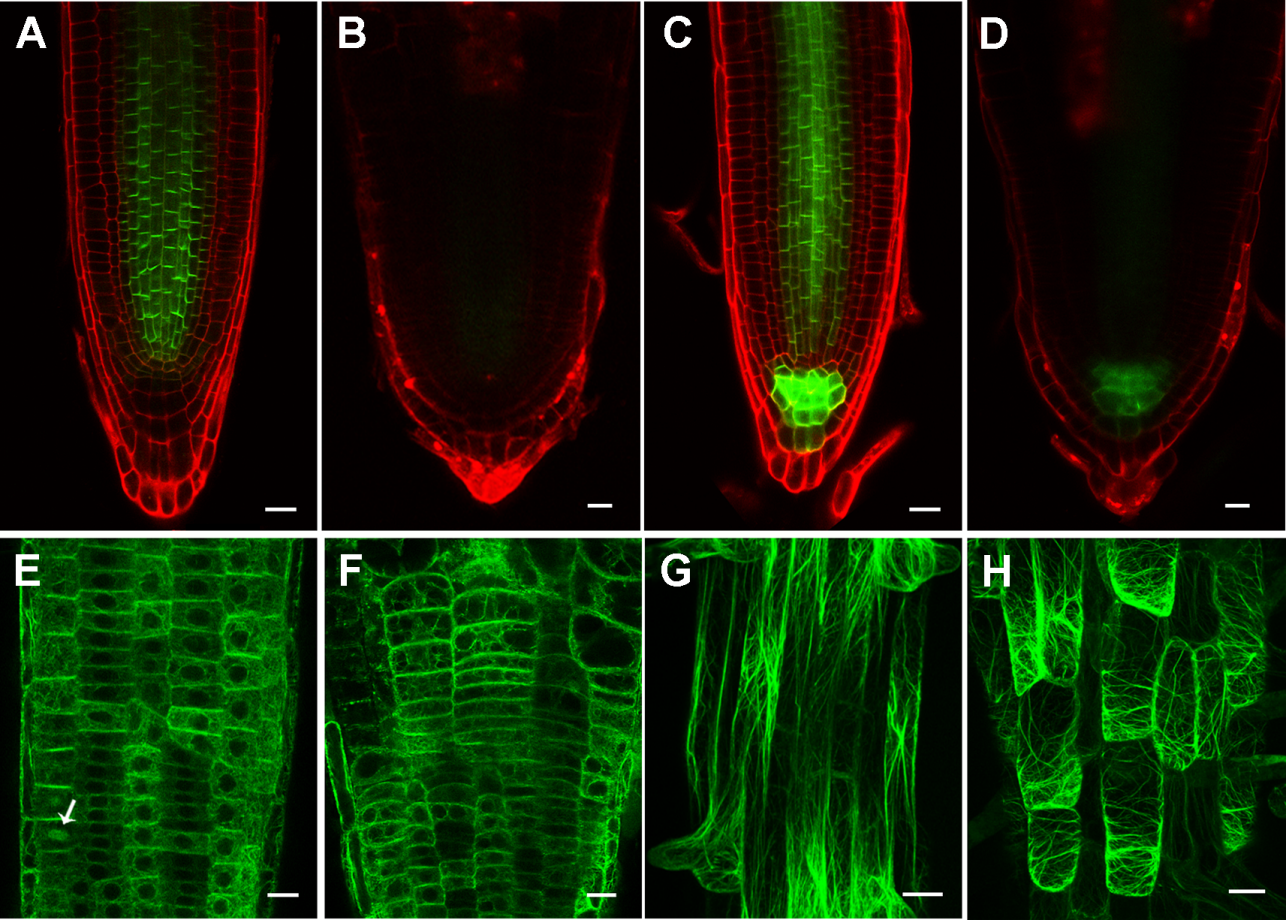
Inhibition of cellulose synthesis by 2,6-dichlorobenzonitrile (DCB) leads to significantly reduced PIN levels, and transversely oriented actin filaments. **(A**, **B)** *PIN1_pro_:PIN1-GFP* expression pattern in 4-day-old wild type root tips treated without **(A)** and with **(B)** 0.1 μM DCB. (c, d) *PIN3_pro_:PIN3-GFP* expression pattern in 4-day-old wild type root tips treated without **(C)** and with **(D)** by 0.1 μM DCB. **(E–H)** Actin filament organization indicated by *35S_pro_ : ABD2-GFP* in 4-day-old wild type root tips **(E, F)** in the meristem zone and **(G, H)** mature zone treated without **(E, G)** and with **(F, H)** 0.1 μM DCB. Arrow indicates phragmoplast-enriched actin filaments. Each figure in **(A, B)** is a maximum projection of five slices z-stack. Bars = 20 μm in (a-f) and 50 μm in (g, h).

To understand how the altered expression of PIN1 and PIN3 and altered actin filament orientation were caused by defective cellulose biosynthesis in *rpi1*, we first tested whether they could be rescued by uridine supplemented into the media.** Indeed, we found both PIN expression (**Figure S10**) and actin orientation (**Figure S11**) were clearly restored by uridine treatment. We further treated wild type seedlings bearing these markers with 0.1 μM of cellulose synthesis inhibitor DCB and observed the expected swelling root tips, significantly reduced PIN levels, and transversely oriented actin filaments (**Figure 7**), which was the same case as in *rpi1*. Treatment with DCB also inhibits primary root growth of these wild type marker lines (**Figure S12**). These results demonstrate the key role of cellulose biosynthesis in maintaining normal expression levels of PIN1 and PIN3, and normal acting actin orientation in the root tips.

## Discussion

### Mutation in *RPI1* Leads to Defective Cellulose Synthesis and Influences Auxin-Dependent Root Development

Several studies showed that the plant cell wall played a key role in auxin-related root development (Zhang et al., 2011; Yang et al., 2014). However, the molecular mechanism underlying remained far from clear. In this work, we further revealed a molecular link between the cell wall and primary root development in *Arabidopsis*. The single-recessive mutant *rpi1* was isolated from our forward genetic screening. The mutant showed significant short and swelling of the primary root and meristem (**Figure 1**). Abnormal cell divisions were observed in the basal region of the embryo from eight-cell to later heart stages (**Figure 2**). These embryonic phenotypes in *rpi1* are similar to those PAT mutants *pin1* and *pin7* (Friml et al., 2003), auguring that auxin-related pathway may function in root development affected by *RPI1* mutation. Indeed, auxin reporter *DR5_rev_:GFP* displayed altered distribution pattern in the *rpi1* roots and embryos (**Figure 3**). Moreover, the mutant showed reduced accumulation of auxin-induced PLT1/PLT2 (**Figure 4**) and misexpression of *MP* (**Figure 4**), which is critical for embryonic root development. In fact, *RPI1* is also one of the many PLT-activated genes (Santuari et al., 2016). Besides, the greatly decreased *WOX5_pro_GFP* (**Figure 1**) suggested that the QC and columella stem cell identity might be affected. Further investigation substantiated that the phenotypes of *rpi1* embryos and seedlings can be attributed to dramatically decreased abundance of auxin efflux carriers PIN1 and PIN3 (**Figure 5**).** Interestingly, the transcription level of *PIN1* and *PIN3* were not obviously affected (**Figure S9**), implying that the reduced abundance of PIN1 and PIN3 could be associated with post-transcription regulated mechanism, such as 26S proteasome (Abas et al., 2006; Kleine-Vehn et al., 2008b; Laxmi et al., 2008) or vacuole-targeted (Abas et al., 2006; Kleine-Vehn et al., 2008b; Laxmi et al., 2008) pathways of protein degradation. Together, our results suggest that RPI1 influences root growth and development through auxin-related pathway.

Impairment of the *RPI1* could result in the decrease in the synthesis of UDP–glucose, a substrate for cellulose synthesis, which eventually led to abnormal cell wall composition in the mutant. Consistent with this, the mutant contains notably lower levels of cellulose compared to the control (**Figure S7**). These phenotypes, combined with the swelling roots, were similar to those observed in *rsw10*, a conditional mutant of *RPI1,* when growing under relatively high temperature (Howles et al., 2006). Besides, the other two radial swelling (rsw) mutants, *rsw1* and *rsw2*, which mutated in the genes encoding cellulose synthase subunit or glycosyltransferase respectively, also showed changes in cellulose levels and short root phenotypes (Arioli et al., 1998; Peng et al., 2000; Lane et al., 2001). Moreover, seedlings grown on the plates containing cellulose synthesis inhibitor, DCB, are almost identical to the root phenotype of *rpi1* seedlings (Peng et al., 2013). These genetic and pharmacological data support the suggestion that the *RPI1* gene does not directly function in the auxin-related pathway but exerts its effects by affecting cellulose synthesis. Interestingly, compared with the *rsw10*, the *rpi1* displayed short and swelling root phenotypes under normal growth conditions, due to their different mutant sites with Glu115Lys in *rsw10* and Ala113Val in *rpi1* (**Figure S2**). The different sensitivity to temperature between these two mutants was probably associated with the different structural changes in RPI1. But the precise mechanism underlying needs to be further addressed. 

### The Role of the Cytoskeleton in PAT and Plant Cell Morphogenesis Regulated by Cell Wall

A previous study has revealed that cell wall functions in regulating PINs localization (Feraru et al., 2011). However, so far, few studies focused on the detailed mechanism underlying interaction between the cell wall and PAT. Several investigations demonstrated an important role of actin cytoskeleton in regulating PIN endocytosis, thus affecting their abundance on the PM (Geldner et al., 2001; Dhonukshe et al., 2008; Nagawa et al., 2012). Moreover, there is evidence showing that defects in cell wall led to abnormal actin arrangement (Zhang et al., 2011; Peng et al., 2013). Given that cell wall defects caused both abnormal actin orientations and reduced PIN protein levels in *rpi1*, it is of high interest to investigate the relationship between actin cytoskeleton reorganization and changes in PIN-dependent polar auxin in future studies.

Given that MTs is the trajectory for cellulose synthesis (McFarlane et al., 2014), most studies on the relationship between cell wall and cytoskeleton showed that defects in wall compositions affect MTs or both MTs and actin filaments organization. Disturbed cortical MT stability and orientation were revealed by genetic and inhibitor analysis (Himmelspach et al., 2003; Chu et al., 2007; Paredez et al., 2008; Peng et al., 2013). Mutation in the *FORMIN HOMOLOGY 5* (*FH5*), an actin-nucleating protein which functions in rice morphology determination, led to abnormal MTs and actin filaments organization (Zhang et al., 2011). Seedlings growing on DCB containing plates also exhibited impairments in both MTs and actin filaments organization, with the actin cytoskeleton only showing changes when treated with high DCB concentration (Peng et al., 2013). In addition to cytoskeleton changes, we also observed reduction in PIN protein accumulation with DCB treatment in wild type plants (**Figure 7**). Compared to previous findings, *rpi1* showed obviously aberrant F-actin orientation (**Figure 6**) whereas quite normal MTs arrangement in root cells (**Figure S8**), suggesting a unique mechanism underlying cell wall regulating cytoskeleton in this mutant.

Besides, it has been believed that actin filaments play fundamental roles in designating a specific division plane during cell division (Rasmussen et al., 2013). Application of actin depolymerizing drugs caused abnormal orientation of division planes in tobacco BY-2 cells (Sano et al., 2005). Loss-of-function mutants of *ACT7* showed defects in division plane orientation (Gilliland et al., 2003). Moreover, the orientation of actin filaments is consistent with the direction of cell elongation in root cells. Collectively, these findings raise a possibility that the transversal F-actin array could be a cause of the abnormal cell division planes observed in *rpi1* mutant root cells (**Figure 1**), which eventually led to the swelling root phenotype.

The influence on actin cytoskeleton from cell wall may be involved in signal transduction. It is reported that mutation in *FEI1* and *FEI2*, two LRR type receptor-like kinase, gives rise to root swelling phenotype which is very similar to *rpi1* (Xu et al., 2008). The *fei1 fei2* showed significantly impaired cellulose synthesis under nonpermissive conditions compared to the wild type. They thought the *FEI* genes may sense the cell wall integrity and then induce the signal transduction to provide a feedback signal for cell wall synthesis (Xu et al., 2008).

In this study, we provide cellular evidence showing the cell wall may act on PAT by modulating actin cytoskeleton. However, the detailed mechanism underlying, for now, is poorly understood. Some researchers argued that the actin-associated formin family proteins might mediate the interplay between the cell wall and actin microfilaments (Liu et al., 2015). This notion is supported by the study of rice type II Formin homology 5 (OsFH5) (Zhang et al., 2011). A report demonstrated that defects in OsFH5 caused abnormal cell wall structure and disorganized cytoskeleton, with actin microfilaments showed more transverse than longitudinal in the root cells (Zhang et al., 2011), suggesting plant formins might play an essential role in cell wall and actin cytoskeleton interaction. Although the actin filament phenotype in *osfh5* is very similar to that in *rpi1*, like most of the studies on cell wall and cytoskeleton, the MTs also severely altered in *osfh5* (Zhang et al., 2011). Investigation for the function of formin proteins in *rpi1* remains to be an attractive subject in the future which will probably clarify the unique mechanism underlying cell wall and actin cytoskeleton interaction.

## Data Availability Statement

All datasets generated for this study are included in the article/**Supplementary Material**.

## Author Contributions

J-BH, YZ, MW, and L-ZT designed the experiments. J-BH, YZ, XZ, MW, and QD performed the experiments. J-BH, YZ, MW, and L-ZT analyzed the data. J-BH and YZ wrote the manuscript. L-ZT revised the manuscript.

## Funding

This research was supported by the grants from National Natural Science Foundation of China (91417316 and 31870280).

## Conflict of Interest

The authors declare that the research was conducted in the absence of any commercial or financial relationships that could be construed as a potential conflict of interest.
