# Supplementary material for: RIBOSE PHOSPHATE ISOMERSASE 1 Influences Root Development by Acting on Cell Wall Biosynthesis, Actin Organization, and Auxin Transport in Arabidopsis
Source: Front Plant Sci. 2020 Jan 8;10:1641. doi: 10.3389/fpls.2019.01641 (PMC6960261; doi:10.3389/fpls.2019.01641)
Supplement: Supplementary file 1 [file DataSheet_1.pdf]

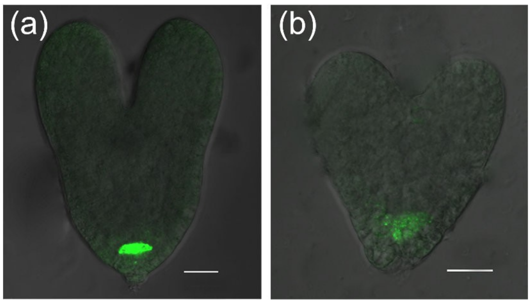

Figure S1. The expression of *WOX5* is affected in the *dpr3* mutant.

(a) The expression of *WOX5$_{pro}$:GFP* in the embryo of wild type.

(b) The expression of *WOX5$_{pro}$:GFP* in the embryo of *dpr3* mutant.

Bars = 20 μm.

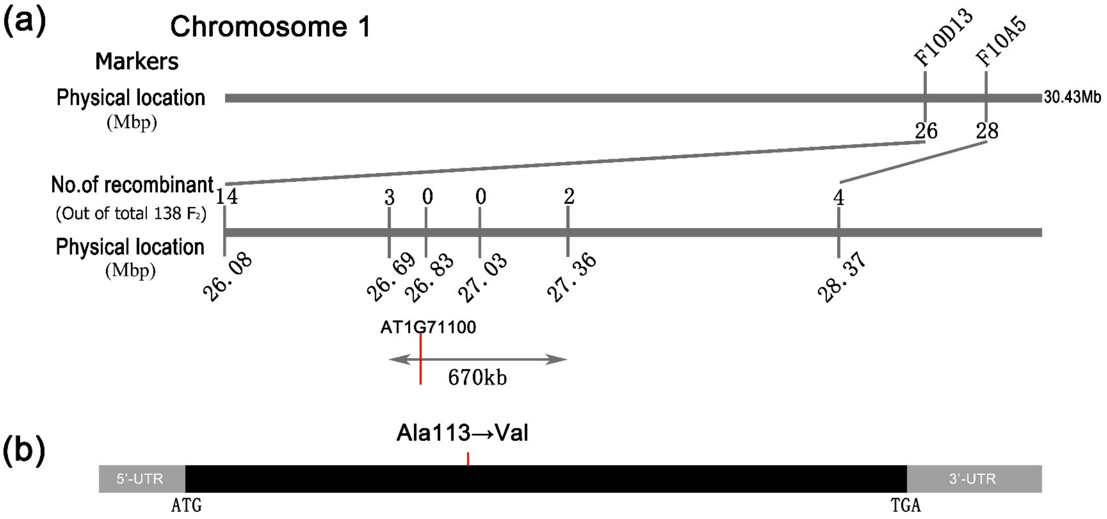

Figure S2. Map-based cloning of *DPR3* gene.

(a) Initial mapping located the *dpr3* mutation to the lower arm of chromosome 1, in a region between the two markers F10D13 and F10A5. Fine mapping located the mutation in a region between 26.69 Mb and 28.37 Mb. Sequencing analysis revealed that a single base pair mutation in the locus AT1G71100 (*RPI1*), leading to a predicted amino acid change Ala113Val.

(b) Gene structure of *RPI1*. The black box represents coding region and gray boxes indicate untranslated regions (UTRs). The vertical line represents the mutation position.

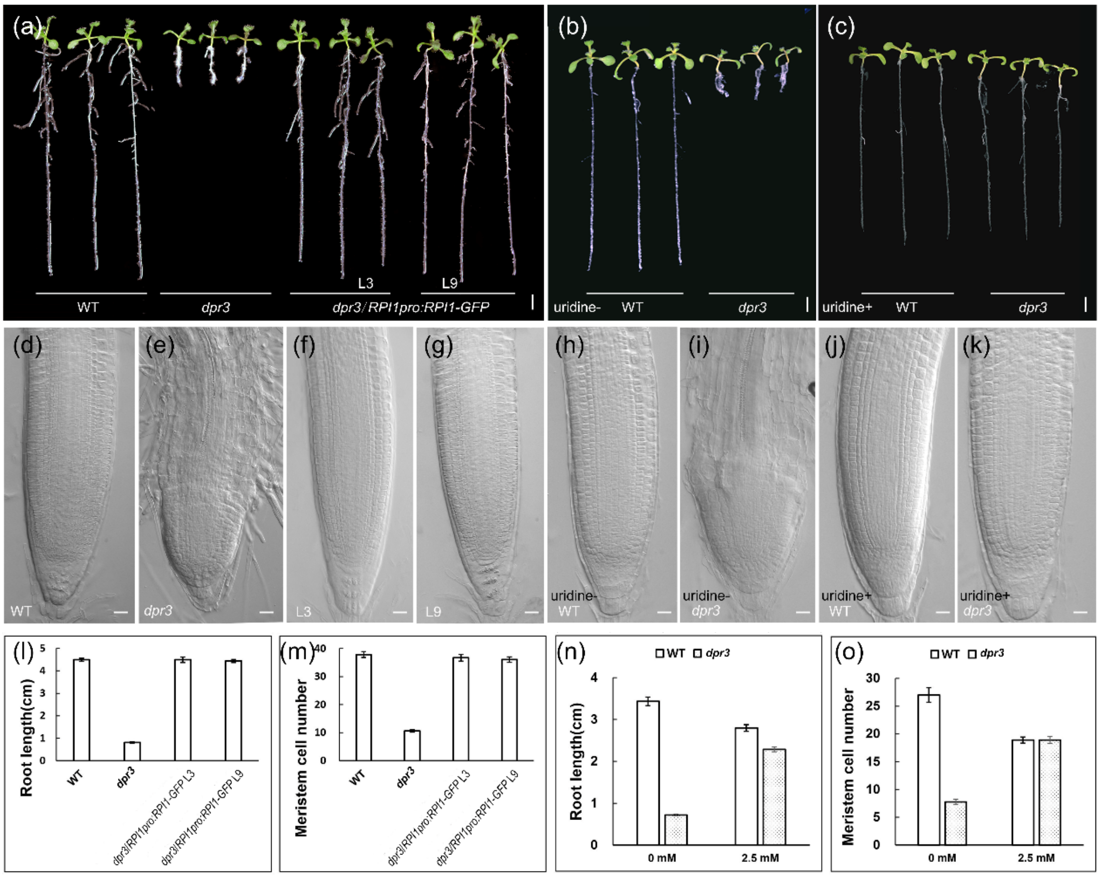

Figure S3. The *RPI1*$_{pro}$*:RPI1-GFP* transgene and exogenous uridine rescue the root phenotypes of *dpr3* mutants.

(a) From left to right, phenotypes of wild type, *dpr3* and *dpr3*/*RPI1*$_{pro}$*:RPI1-GFP* transgenic (L3, L9 lines) seedlings at 10 DAG.

(b, c) Phenotypes of wild type (left) and *dpr3* (right) at 9 DAG without (b) and with (c) 2.5 mM uridine supplemented in the plates.

(d–g) Root tips of the wild type (d), *dpr3* (e) and *dpr3*/*RPI1*$_{pro}$*:RPI1-GFP* transgenic plants (L3, L9 lines) at 10 DAG.

(h-k) Root tips of the wild type (h) and *dpr3* (i) seedlings at 9 DAG without (h,i) and with (j,k) 2.5 mM uridine supplemented in the plates.

(l) Primary root lengths of the wild type, *dpr3*, and *dpr3*/*RPI1*$_{pro}$*:RPI1-GFP* transgenic plants (lines L3 and L9) at 10 DAG. Data shown are average and SD (n = 32).

(m) Meristem cell number of the indicated genotypes at 10 DAG. Data shown are average and SD (n = 28).

(n) Primary root lengths of the indicated plants without or with 2.5 mM uridine at 9 DAG.

Data shown are average and SD (n = 30).

(o) Meristem cell number of indicated plants without or with 2.5 mM uridine at 9 DAG (n = 30).

Bars = 5 mm in (a-c) and 20 μm in (d-k).

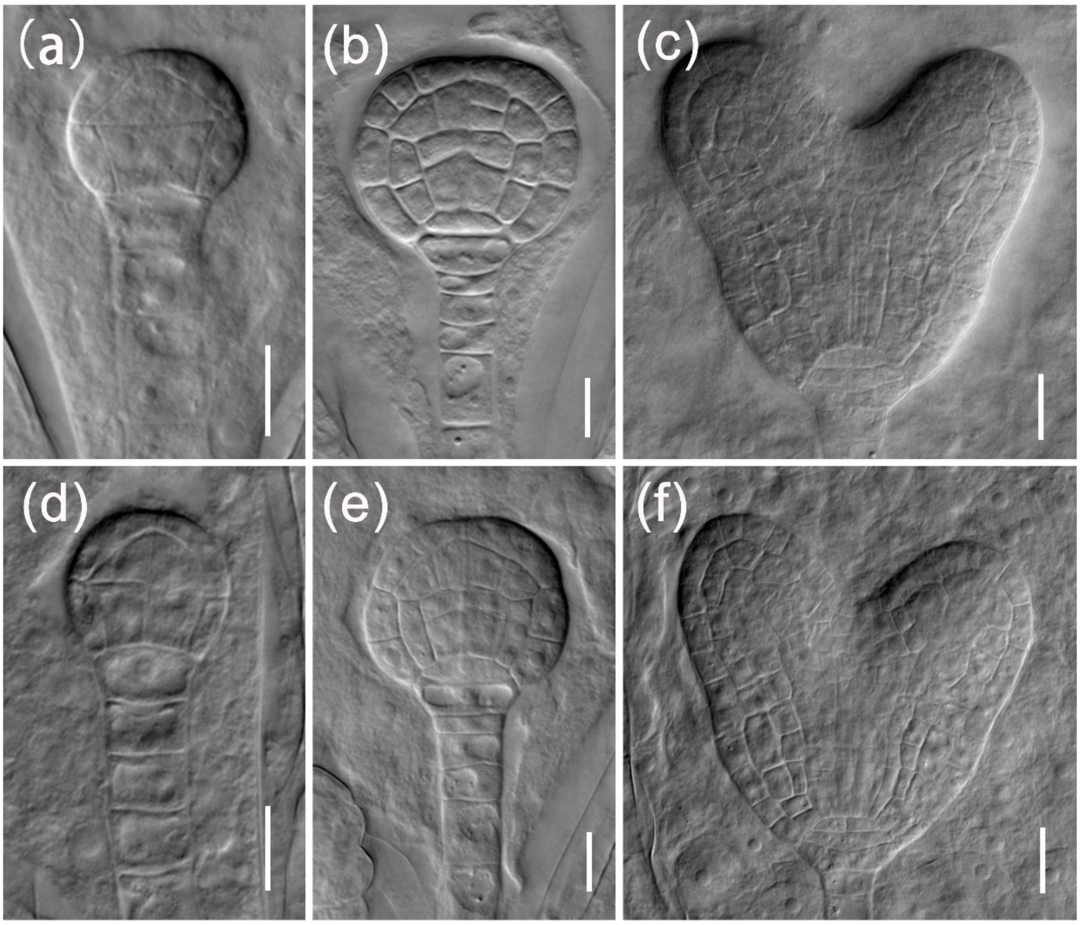

Figure S4. The *RPI1*$_{pro}$*:RPI1-GFP* transgene rescue the embryo phenotypes of *dpr3* mutants.

(a–c) Wild type embryos at 16-cell (a), globular (b) and heart (c) stages.

(d–f) Embryos of *dpr3*/*RPI1*$_{pro}$*:RPI1-GFP* transgenic plants at 16-cell (d), globular (e) and heart (f) stages.

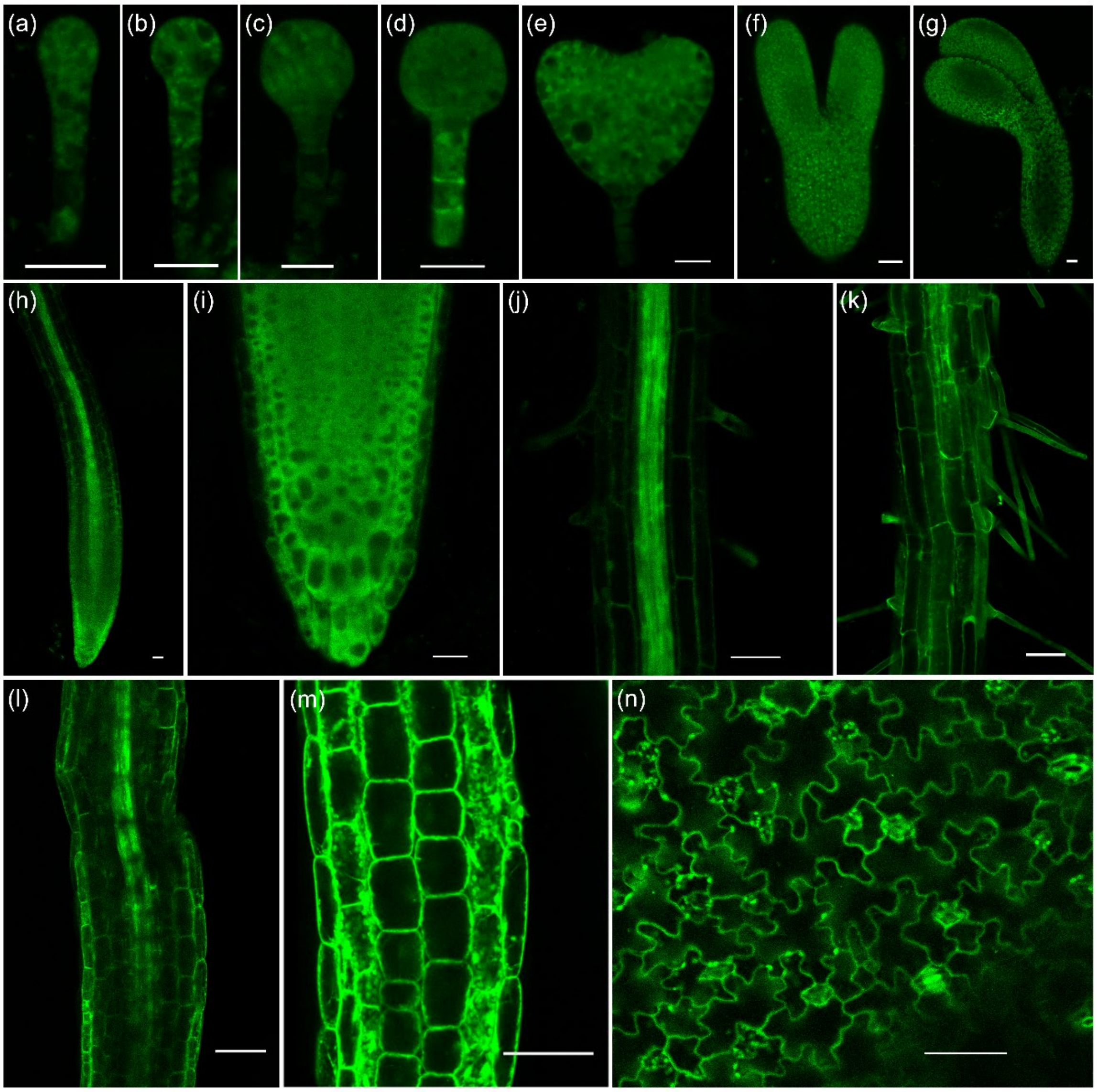

Figure S5. *RPI1* expression pattern and subcellular localization.

(a–g) *RPI1pro:RPI1-GFP* expression pattern in the embryos from octant to bent stages. **(h–k)** *RPI1pro:RPI1-GFP* is expressed in the root tip (h) and mature zone (j, k). RPI1-GFP is localized in the cytoplasm of the root cells (i).

(l, m) *RPI1pro:RPI1-GFP* is expressed in the hypocotyl.

(n) *RPI1pro:RPI1-GFP* is expressed in pavement and guard cells of cotyledon.

Bars = 20 μm.

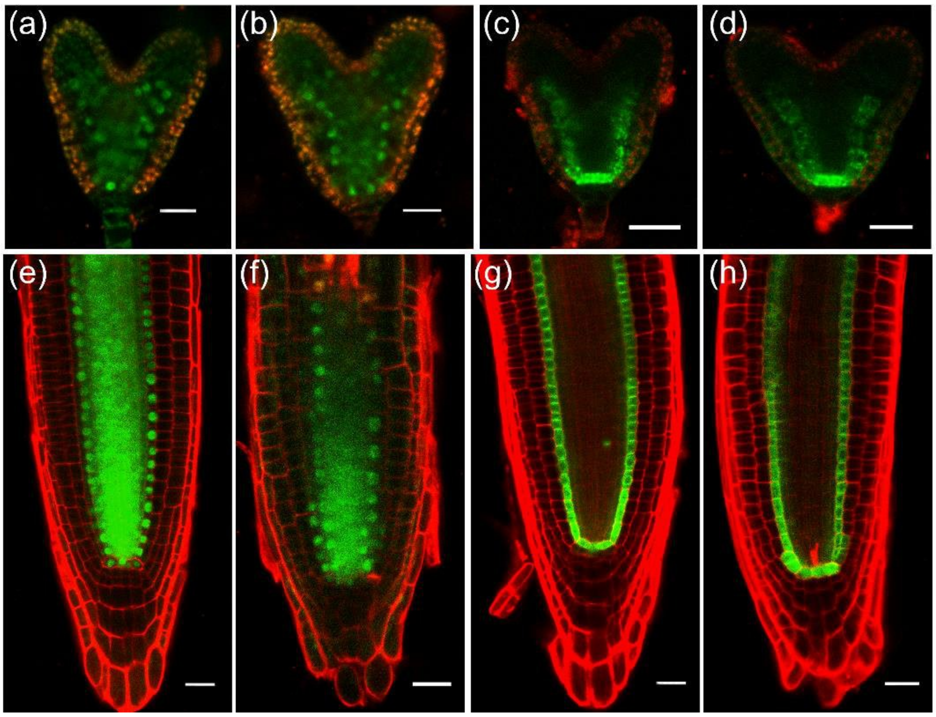

Figure S6. *RPI1* does not affect the expression of *SHR* and *SCR* in embryos and roots.

(a, b) *SHR$_{pro}$:SHR-GFP* expression in wild type (a) and *rpi1* (b) embryos.

(c, d) *SCR$_{pro}$:GFP* expression in wild type (c) and *rpi1* (d) embryos.

(e, f) *SHR$_{pro}$:SHR-GFP* expression in the roots of 4-d-old wild type (e) and *rpi1* (f) seedlings.

(g, h) *SCR$_{pro}$:GFP* expression in the roots of 4-d-old wild type (g) and *rpi1* (h) seedlings.

Bars = 20 μm.

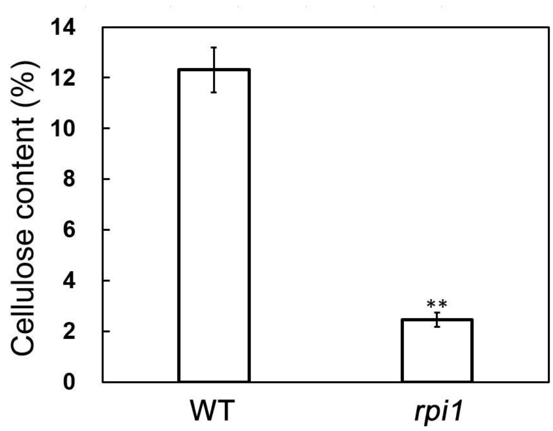

Figure S7. Cellulose content is decreased in the *rpi1* mutant.

Cellulose content is calculated as the percentage of cellulose amount relative to dry weight of 8-d-old seedlings. Error bars indicate ±SD of three biological replicates. Asterisks indicate statistically significant differences in cellulose content between *rpi1* mutant and WT determined by Student's *t*-test (** $p < 0.01$).

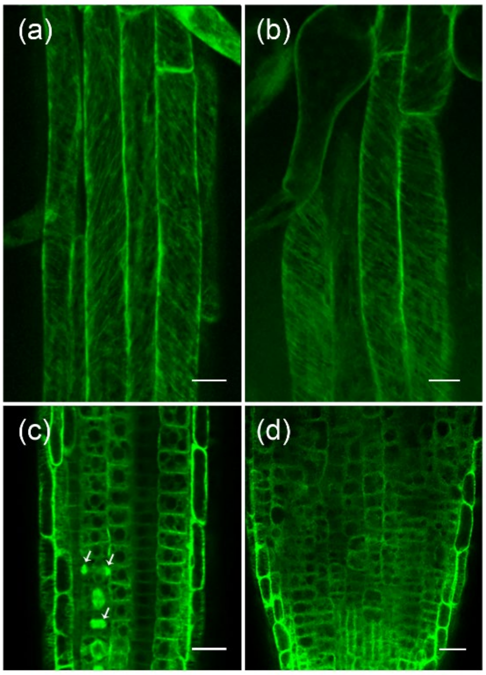

Figure S8. Microtubule arrays are not affected in *rpi1* mutant.

(a-b) *35S$_{pro}$:GFP-tubulin* expression pattern in root mature zone of 4-d-old (a) wild type (b) *rpi1* seedlings.

(c-d) *35S$_{pro}$:GFP-tubulin* expression pattern in root mature zone of 4-d-old (c) wild type (d) *rpi1* seedlings. Arrows indicate phragmoplast-enriched microtubules.

Bars = 20 μm.

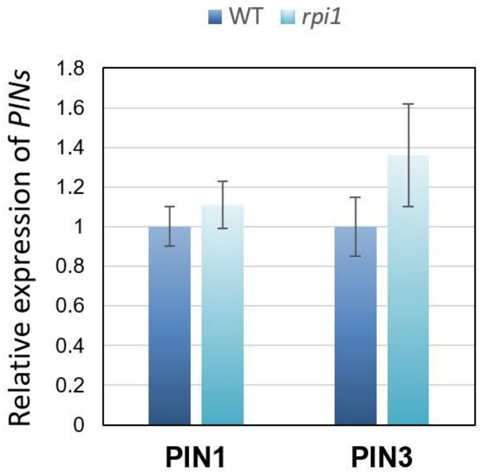

Figure S9. The mRNA levels of *PIN1 and PIN3* are not significantly affected in *rpi1* mutant. Relative expression of *PIN1* and *PIN3* in 5-d-old wild type (WT) and *rpi1* roots, as determined by qRT-PCR. Data represent means±SD (n=3).

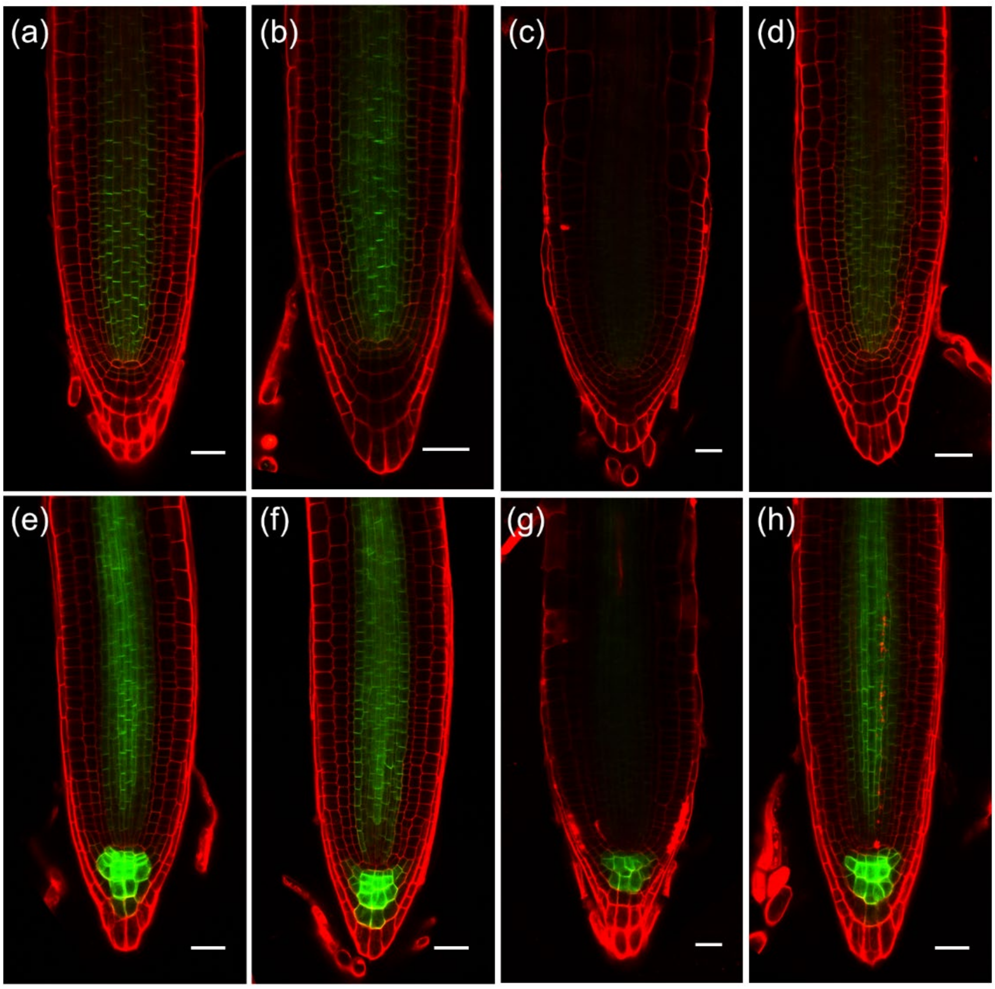

Figure S10. The expression of *PIN1*$_{pro}$*:PIN1-GFP* and *PIN3*$_{pro}$*:PIN3-GFP* in *rpi1* mutant could be rescued by uridine.

(a-d) *PIN1*$_{pro}$*:PIN1-GFP* expression pattern in 4-d-old (a, b) wild type and (c, d) *rpi1* root tips (b, d) treated without (a, c) and with (b,d) 2.5 mM uridine.

(e-h) *PIN3*$_{pro}$*:PIN3-GFP* expression pattern in 4-d-old (e, f) wild type and (g, h) *rpi1* root tips (f, h) treated without (e, g) and with(f, h) 2.5 mM uridine.

Bars = 20 μm.

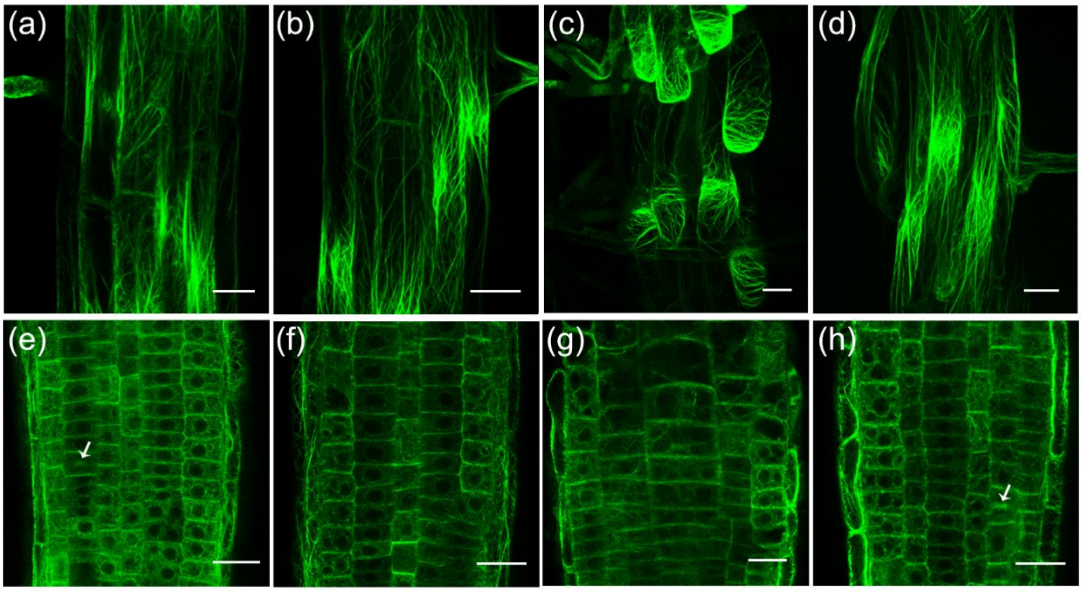

Figure S11. Actin filament organization in in *rpi1* mutant was rescued by uridine.

(a-h) Actin filament organization indicated by *35S$_{pro}$:ABD2-GFP* in 4-d-old (a, b, e, f) wild type and (c, d, g, h) *rpi1* root tips (e-h) in the meristem zone and (a-d) mature zone (b, d, f, h) treated without (a, c, e, g) and with (b, d, f, h) 2.5 mM uridine. Arrows indicate phragmoplast-enriched actin filaments. Each figure in (a-d) is a maximum projection of 5 slices z-stack.

Bars = 50 μm in (a-d) and 50 μm in (e-h).

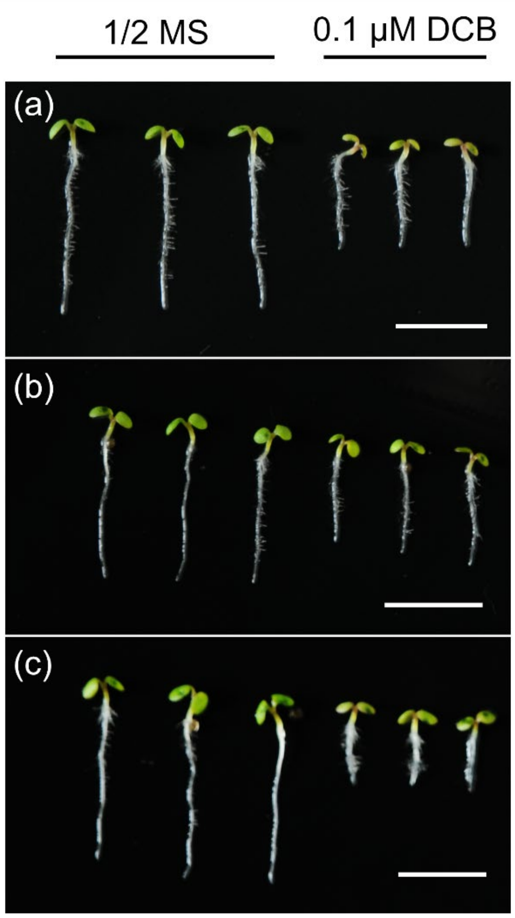

Figure S12. 2,6-dichlorobenzonitrile (DCB) treatment inhibits root growth of wild type seedlings bearing *PIN1*$_{pro}$*:PIN1-GFP*, *PIN3*$_{pro}$*:PIN3-GFP* and *35S*$_{pro}$*:ABD2-GFP* markers.

(a-c) Primary root length of 4-d-old wild type seedlings expressing (a) *PIN1*$_{pro}$*:PIN1-GFP*, (b) *PIN3*$_{pro}$*:PIN3-GFP* and (c) *35S*$_{pro}$*:ABD2-GFP* treated by 0.1 μM DCB.

Bars = 5 mm.

Table S1. Primers used in this study.

Map-based cloning	F10D13-F<br>F10D13-R	5'- GTGGTTGCCTACAGCTTTA -3'<br>5'- AGAAGAATGACGACAATAGT -3'
	F15H11-F<br>F15H11-R	5'- GTGAAAACTGAAACGTATGT -3'<br>5'- TACCGGCCTCAATTTGAC -3'
	F23N20-F<br>F23N20-R	5'- TTGTTTTCCATTTGGTTAG -3'<br>5'- TTTGATGGTCGTGTTTAAG -3'
	F17M19-F<br>F17M19-R	5'- GTTTTTGTGGGGGAATCA -3'<br>5'- CAATCAGAACCAGACTCATA -3'
	F28P22-F<br>F28P22-R	5'- TGTTTTTTAGGACAAATGGCG -3'<br>5'- CTCCAGTTGGAAGCTAAAGGG -3'
	F10A5-F<br>F10A5-R	5'- CAAAACTCCCATCTTGCTTCA -3'<br>5'- CCGGTGGATATCGACTTAGG -3'
Vector construction	$RPI1_{pro}$-F<br>$RPI1_{pro}$-R	5'- CTCACCGTTGAGACTACTG -3'<br>5'- TGTACGCGACTTGCTTTGTGA -3'
	RPI1 cDNA-F<br>RPI1 cDNA -R	5'- ATGGGTTCTGCATTCGATCCCC -3'<br>5'- TCCAAACCTATCCTTGACGGTAACA -3'
qRT-PCR	PIN1-F<br>PIN1-R	5'-TGGAAGACAACCTTTGGAAACT-3'<br>5'-TGAAGCATTAGAACGACGAACA-3'
	PIN3-F<br>PIN3-R	5'-CAAGTGGAGATTTCGGAGGA-3'<br>5'-GCGTCTTTTGGTCTCTCTGC-3'

Table S2. Quantitative analysis of *rip1* embryonic defects at indicated developmental stages.

Stage / Genotype	16-32 cell	Globular	Heart	Defect/Total	Percentage
WT	2/115 (1.73%)	3/259 (1.16%)	2/224 (0.89%)	7/598	1.17 %
rpi1	25/312 (8.01%)	51/462 (11.04%)	42/341 (12.32%)	118/1115	10.58 %
rpi1/RPI1$_{pro}$:RPI1:GFP	2/98 (2.04%)	2/212 (0.94%)	2/185 (1.08%)	6/495	1.21 %
